# Mesoderm is required for coordinated cell movements within zebrafish neural plate *in vivo*

**DOI:** 10.1186/1749-8104-9-9

**Published:** 2014-04-23

**Authors:** Claudio Araya, Marcel Tawk, Gemma C Girdler, Marta Costa, Carlos Carmona-Fontaine, Jonathan DW Clarke

**Affiliations:** 1 Medical Research Council (MRC) Centre for Developmental Neurobiology, King’s College London, New Hunt’s House, 4th Floor, Guy’s Hospital Campus, London SE1 1UL, UK; 2 Laboratory of Developmental Biology, Instituto de Ciencias Marinas y Limnológicas, Facultad de Ciencias, Universidad Austral de Chile, Campus Isla Teja s/n, Valdivia 5090000, Chile; 3 Hôpital Kremlin Bicêtre, 80 Rue du General Leclerc, Le Kremlin Bicêtre 94276, France; 4 Medical Research Council (MRC) Laboratory of Molecular Biology (LMB), Francis Crick Avenue, Cambridge Biomedical Campus, Cambridge CB2 0QH, UK; 5 Department of Zoology, University of Cambridge, Downing Street, Cambridge CB2 3EJ, UK; 6 Program in Computational Biology, Memorial Sloan-Kettering Cancer Center, New York, NY 10065, USA

**Keywords:** Zebrafish neurulation, Morphogenesis, Mesoderm

## Abstract

**Background:**

Morphogenesis of the zebrafish neural tube requires the coordinated movement of many cells in both time and space. A good example of this is the movement of the cells in the zebrafish neural plate as they converge towards the dorsal midline before internalizing to form a neural keel. How these cells are regulated to ensure that they move together as a coherent tissue is unknown. Previous work in other systems has suggested that the underlying mesoderm may play a role in this process but this has not been shown directly *in vivo*.

**Results:**

Here we analyze the roles of subjacent mesoderm in the coordination of neural cell movements during convergence of the zebrafish neural plate and neural keel formation. Live imaging demonstrates that the normal highly coordinated movements of neural plate cells are lost in the absence of underlying mesoderm and the movements of internalization and neural tube formation are severely disrupted. Despite this, neuroepithelial polarity develops in the abnormal neural primordium but the resulting tissue architecture is very disorganized.

**Conclusions:**

We show that the movements of cells in the zebrafish neural plate are highly coordinated during the convergence and internalization movements of neurulation. Our results demonstrate that the underlying mesoderm is required for these coordinated cell movements in the zebrafish neural plate *in vivo*.

## Background

Morphogenesis of the vertebrate neural tube from the neural plate is a fundamental early step in building the brain and spinal cord. This complex process is likely to be coordinated by a combination of mechanisms both intrinsic and extrinsic to the neural tissue itself. One important intrinsic mechanism is the non-canonical Wnt/planar cell polarity (PCP) pathway that regulates the movements of convergent extension to shape the neural plate. This pathway is thought to act through cell-cell interactions within the neural plate itself and appears to be a prerequisite for efficient neural tube closure and morphogenesis in all vertebrates (reviewed by Ueno and Greene [1]). However, embryological and genetic approaches have also suggested that adjacent tissues such as the mesoderm can directly influence neural tube morphogenesis [2-5]. Mutations affecting proliferation in mouse cephalic mesoderm suggest that this tissue is critical for the correct shape and closure of anterior neural tube structures [4] (reviewed by Copp *et al*. [6]). In addition, experiments using amphibian explant cultures suggest that persistent interactions with the underlying mesoderm are required for the cell elongation, cell protrusive and cell intercalation events present during neural plate morphogenesis *in vitro*[7,8]. However, the degree to which such tissue interactions influence cell behavior has not been analyzed *in vivo*.

The zebrafish embryo provides a good model to study tissue interaction *in vivo* because of its superior optical qualities. A common feature in teleost and other vertebrate embryos is that the neural plate lies on a subjacent layer of mesoderm and the first steps in the process of neurulation involve the convergence of the neural plate towards the dorsal midline [9,10]. The later stages of neurulation in teleost embryos are different to other vertebrates in that the neural tube is not formed by folding an epithelial neural plate, rather the teleost neural tube is built by generating a lumen at the center of a solid neural rod primordium (reviewed by Lowery and Sive [11] and Clarke [12]). The solid neural rod is built by the orchestrated actions of large numbers of cells from both sides of the neural plate that converge towards the dorsal midline where they become internalized. The mechanism of neural internalization in the teleost is a poorly understood process but it results in a structure known as the neural keel, which then condenses into a solid neural rod. Subsequently, the neural rod cavitates to form a neural tube with a single central lumen surrounded by neuroepithelium with clear apicobasal polarity. At a cellular level, neural tube architecture is achieved by a combination of behaviors including cell intercalation, midline-crossing divisions and polarized cell behavior [10,13-18]. A possible role for mesoderm in zebrafish neurulation is suggested by the anterior brain defects in maternal-zygotic *one-eyed pinhead* (MZ*oep*) mutant embryos, which lack Nodal signaling and anterior mesoderm [19], but a detailed analysis of neural morphogenesis in these mutants is lacking.

To better understand the roles of mesoderm in neural tube morphogenesis we have taken a live imaging approach to analyze neural cell movements in normal embryos and embryos lacking mesoderm. We found that at early stages of zebrafish neurulation the underlying mesoderm is required for the coordinated movements of neural plate cells. In the absence of mesoderm a neural primordium does develop at the dorsal midline but its tissue architecture is severely disorganized.

## Results

### MZ*oep* embryos have severe defects in neural tube morphogenesis

**Figure 1:**
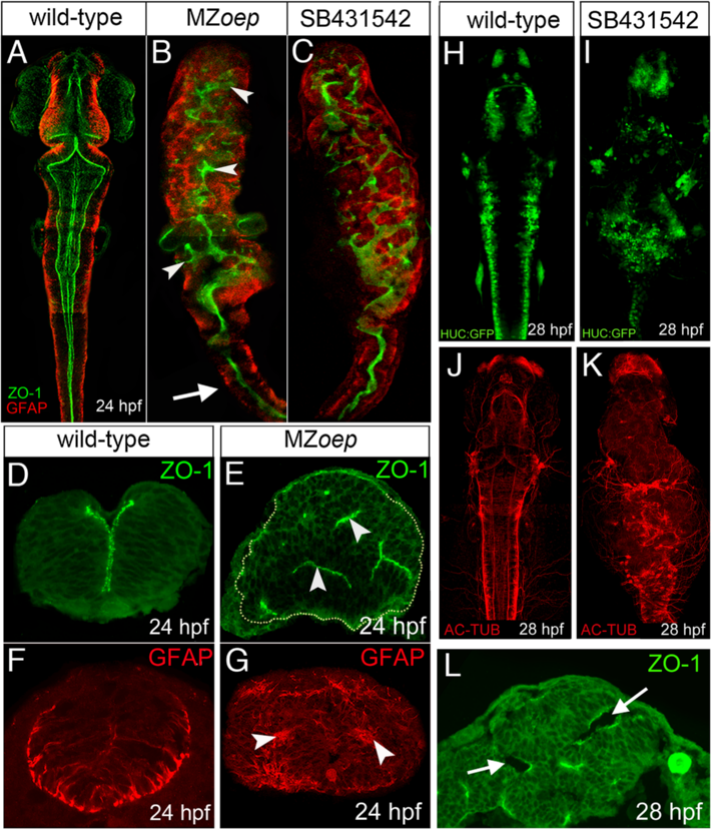
**MZ*oep *mutants have aberrant neural tube organization. (A)** Projection of confocal z-series showing dorsal view of brain and anterior spinal cord. The midline ventricle is lined by ZO-1 expression (green) and the basal regions of the neuroepithelium are lined by the GFAP expression (red). **(B)** Projection of confocal z-series showing dorsal view of brain and anterior spinal cord from MZ*oep* mutant. Both apical ZO-1 and basal GFAP expression show extensive disruption to neural tube morphology in brain regions (arrowheads) but appear relatively normal in anterior spinal cord (arrow). **(C)** Projection of confocal z-series showing dorsal view of brain and anterior spinal cord from embryo treated with the Nodal inhibitor SB-431542. **(D,E)** Transverse sections show the normal single midline domain of ZO-1 appears discontinuous and more randomly oriented in MZ*oep* embryo. **(F,G)** The basal marker GFAP is expressed in ectopic foci deep from the surface of the MZ*oep* neural primordium. **(H,I,J,K)** Neurons labeled with tg(HUC-GFP) (green) and their axons labeled with Ac-tub (red) are present but disorganized in the Nodal-defective embryo brains. **(L)** By 28 hpf ectopic ventricles (arrowed) have opened up in the MZ*oep* brains. Ac-tub, anti-acetylated tubulin antibody; GFAP, glial fibrillary acidic protein; hpf, hours post fertilization; MZ*oep*, maternal-zygotic *one-eyed pinhead*; ZO-1, zonula occludens 1.

To assess the role of the mesoderm during neural tube formation, we directly compared neuroepithelial organization between wild-type and MZ*oep* embryos, which lack Nodal signaling and mesoderm derivatives in the head [20,21]. By 24 hours post fertilization (hpf), wild-type brains show a well-organized ventricle revealed by the apical protein zonula occludens 1 (ZO-1) at the ventricular surfaces of the neural tube (Figure 1A), while the cytoskeletal protein glial fibrillary acidic protein (GFAP) is concentrated in the basal end feet of neuroepithelial cells at the perimeter of the neural tube (Figure 1A). In contrast, MZ*oep* mutants have severely disrupted ZO-1 expression revealing a disorganized ventricular surface (Figure 1B), including out-pockets and apparently isolated domains of ZO-1. The MZ*oep* phenotype is reproduced by treating wild-type embryos with the Nodal inhibitor SB-431542 from the one-cell stage (Figure 1C) [22]. Transverse sections through the hindbrain region at 24 hpf confirm the existence of multiple ectopic ZO-1 foci in the MZ*oep* brains (Figure 1D,E) and GFAP staining is also abnormal and no longer restricted to the perimeter of the neural tube (Figure 1F,G). Despite its abnormal architecture, by 28 hpf the neural primordium is still able to generate neurons and axons (Figure 1H,I,J,K), as well as ventricular spaces (Figure 1L), demonstrating that several fundamental properties of the neuroepithelium are still intact.

**Figure 2:**
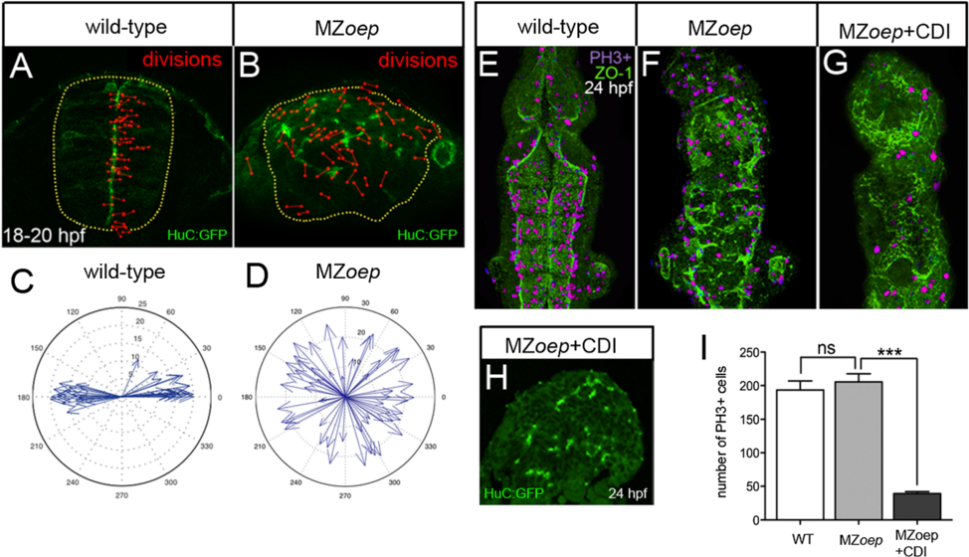
**Ectopic divisions do not generate the abnormal neural tube in MZ*oep *embryos. (A)** Location and orientation of divisions monitored over a 2-hour time-lapse period of neural rod development in wild-type embryo by analyzing the expression of apical marker Pard3-GFP (green) and H2B-RFP mRNA to label nuclei (not included in image for clarity, red dumbbells indicate location and orientation of dividing cells). Wild-type neural progenitor divisions are strongly orientated along the mediolateral axis of the developing neural tube. Yellow dots outline the rod. **(B)** Location and orientation of divisions monitored over a 2-hour time-lapse period of neural rod development in MZ*oep* embryo. **(C,D)** Orientation plots of divisions in wild-type and MZ*oep* embryos. **(E,F,G)** Dorsal projections of ventricle morphology (ZO-1, green) at 24 hpf in wild-type, MZ*oep* embryos and MZ*oep* embryos treated with CDIs (hydroxyurea and aphidicolin). Blocking division does not rescue ventricle morphology. PH3 staining of mitotic figures (purple) used to calculate efficiency of division block. **(H)** Transverse section of brain from MZ*oep* embryo treated with division blockers. **(I)** Quantification of divisions in wild-type, MZ*oep* and MZ*oep* division blocked embryos. Number of cell divisions between wild-type 194 and MZ*oep* 206, *P* = 0.5129 Student’s *t*-test and between MZ*oep* 206 and MZ*oep* + CDI 39, ***P <0.0001 Student’s *t*-test. In **(E)**, **(F)** and **(G)**, equivalent tissue volumes were used (360 μm in length x 130 μm in width x 90 μm in depth). In **(I)**, error bars indicate SEM. CDI, cell division inhibitor; H2B-RFP, histone H2B/red fluorescent protein fusion; hpf, hours post fertilization; MZ*oep*, maternal-zygotic *one-eyed pinhead*; Pard3-GFP, green fluorescent protein/polarity protein partitioning defective 3 fusion; PH3, phospho-histone 3 marker; SEM, standard error of the mean; wt, wild-type; ZO-1, zonula occludens 1.

In wild-type embryos, stereotyped cell divisions normally occur close to the midline of the developing neural rod [23,24,13] and these have been shown to orchestrate cellular organization and ventricle formation at neural midline [14-16]. To test whether the disorganized ventricles in MZ*oep* mutants might result from disruptions to the midline divisions, we monitored division location and orientation by time-lapse confocal microscopy. We found that neural divisions were both misoriented and ectopic in MZ*oep* mutant embryos (Figure 2A,B,C,D). It is possible that the misoriented and ectopic divisions contribute to the development of the disorganized lumen; however, the alteration in these divisions is not the primary cause of the neural tube defects here, because in contrast to the situation in other mutants with disrupted divisions [13-16] blocking cell division in MZ*oep* mutants does not rescue neural tube morphology (Figure 2E,F,G,H,I).

**Figure 3:**
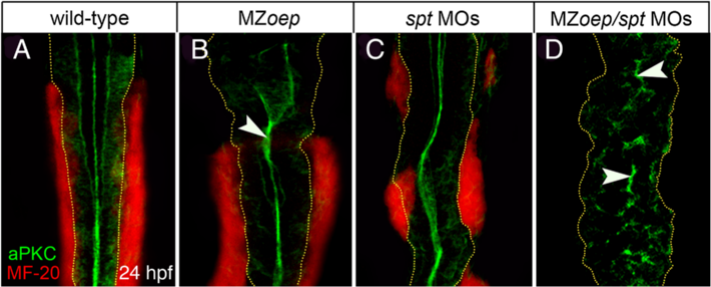
**Lack of mesoderm disrupts neural tube morphogenesis in both anterior and posterior levels of the neuro**-**axis. (A,B)** Projection of confocal z-series showing dorsal view of mesoderm (red), expressing the muscle marker (MF-20) and aPKC (green) in caudal hindbrain and anterior spinal cord at 24 hpf. The normal midline domain of aPKC is maintained in regions of MZ*oep* embryo that are adjacent to mesoderm (arrowhead). **(C)** Mesoderm is still present but disrupted in *spt*-deficient embryos and the aPKC domain is largely normal. **(D)** Embryos deficient for both *oep* and *spt* have no mesoderm adjacent to the spinal cord and apical aPKC is severely disrupted in spinal cord (arrowheads). Basal extremity of neural tube indicated by yellow dotted lines. aPKC, atypical protein kinase C; hpf, hours post fertilization; MZ*oep*, maternal-zygotic *one-eyed pinhead*.

Examination of neural organization along the anterior-posterior axis of MZ*oep* mutants showed the defect was not present at all axial levels. In the brain, neuroepithelial organization is disrupted in fore-, mid- and hindbrain levels (Figure 1B). However, in the spinal cord region the apical marker atypical protein kinase C (aPKC) is present in a single midline domain similar to wild-type spinal cord (Figure 3A,B). Staining for the skeletal muscle marker MF-20 reveals this normal organization of the neural midline is coincident with the presence of somitic mesoderm adjacent to the neural tissue in the MZ*oep* embryos (Figure 3B). To ask whether the presence of these mesodermal derivatives is responsible for the normal spinal cord organization, we analyzed *spt* embryos that have disrupted posterior mesoderm and MZ*oep*/*spt* embryos that completely lack both anterior and posterior mesoderm [25]. As previously described for *spt* mutants [25], wild-type embryos injected with *spt* morpholino retain intermittent patches of MF-20 staining adjacent to the spinal cord but this mesoderm is not continuous as it is in wild-type embryos (Figure 3C). However, in MZ*oep* embryos injected with *spt* morpholino, we found that neural organization is severely disrupted both in the brain (not shown) and spinal cord (Figure 3D). These observations suggest that the adjacent mesoderm may be a critical tissue for organizing neural tube morphogenesis at all anterior-posterior levels.

### The mesoderm, but not Nodal signaling, is required for zebrafish neurulation

**Figure 4:**
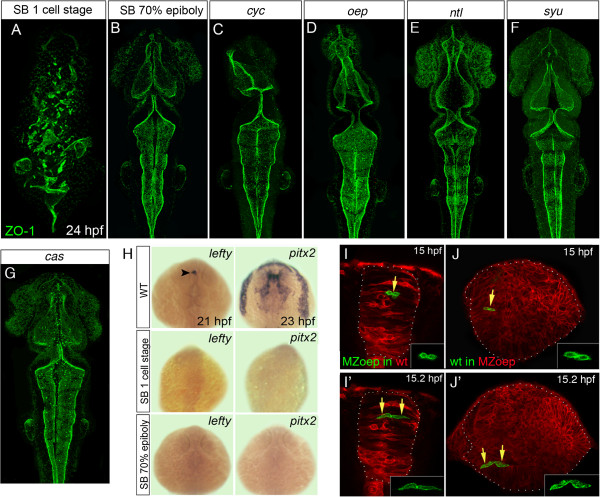
**Loss of mesoderm, but not Nodal signaling, leads to neural tube defects. (A,B,C,D,E,F,G)** Projection of confocal z-series showing dorsal view of 24 hpf zebrafish embryos staining for the apical marker ZO-1 (green). **(A)** Disrupted ventricular organization of embryo treated with the Nodal inhibitor SB-431542 from the one-cell stage. **(B)** Treatment with SB-431542 from 70% epiboly does not cause neural tube defects. **(C,D)** Ventricle organization in Nodal mutants *cyc* and *oep* is largely normal. **(E,F)** Ventricle organization in *ntl* and *syu* mutants is normal. **(G)** Ventricle organization in the endoderm mutant *cas* mutants is normal. **(H)** Expression of the Nodal-dependent markers *lefty* and *pitx2* show the SB-431542 drug is an efficient blocker of Nodal signaling. All embryos between 21 and 23 hpf, dorsal view. Arrowhead in **(H)** indicates asymmetric *lefty* expression at 21 hpf. **(I,I’)** Two frames from time-lapse sequence show transplanted MZ*oep* cells (green) integrate and divide to make mirror-image daughters just like host cells in a wild-type embryo at 15 hpf. **(J,J’)** Two frames at 15 hpf from time-lapse sequence show wild-type cells (green) divide to make mirror-image daughters after transplantation to a MZ*oep* embryo. Unlike divisions in wild-type embryos, however, these divisions can be far from their normal location at the midline. hpf, hours post fertilization; MZ*oep*, maternal-zygotic *one-eyed pinhead*; wt, wild-type; ZO-1, zonula occludens 1.

*Oep* is an essential co-factor for Nodal signaling and required for mesoderm/endoderm development [20,21]. Therefore, the disruption to neural tube organization in MZ*oep* embryos could result from loss of Nodal signaling rather than mesoderm. To test this, we blocked Nodal signaling just prior to neurulation in wild-type embryos by using the inhibitor SB-431542, which effectively inhibits Nodal signaling by blocking the kinase activity of ALK4, 5 and 7 receptors [22]. Wild-type embryos raised in SB-431542 from the two- to four-cell stage display the same abnormal neural tube phenotype as MZ*oep* (Figure 4A). However, embryos treated with SB-431542 from just prior to neurulation (from 70 to 80% epiboly, 7 to 8 hpf) develop with normal neural tube morphogenesis (Figure 4B). We extended this analysis to other Nodal-deficient embryos such *cyclops* (*cyc*), which lack floorplate [26], and zygotic *oep*, which have defective notochord and lack floorplate [20], and found that they also developed well-organized neural tubes (Figure 4C,D). To further address whether midline defects might be a factor in neural tube disorganization we also analyzed *no*-*tail* and *sonic you* mutants. Both developed a normal midline ventricle (Figure 4E,F) indicating that the loss of midline structures or signaling is not the cause of neural defects in MZ*oep* embryos. Finally we examined *casanova* (*cas*) mutant embryos lacking head endoderm and they also developed normal ventricle morphology (Figure 4G).

To test the efficacy of our SB-431542 treatment, we examined the expression of the Nodal downstream genes *lefty1*[27] and *pitx2*[28]. We found the wild-type expression of both *lefty1* and *pitx2* (Figure 4H) was lost in embryos treated with SB-431542 (Figure 4H) at either the one-cell stage or just prior to neurulation (70 to 80% epiboly).

To test the cell autonomy of neural plate cell behavior in the MZ*oep* embryos we carried out cell transplantation experiments. MZ*oep* cells transplanted into a wild-type background were not only able to incorporate into the wild-type neural plate with a normal elongated morphology, but also undergo midline-crossing divisions at the same time as their wild-type peers (Figure 4I) and generate a pair of daughter cells of mirror-symmetric morphology (Figure 4I’). In complementary experiments, although wild-type cells transplanted into MZ*oep* background followed the abnormal movements of mutant neural primordium, wild-type cells were able to divide and generate a pair of mirror-image sister cells within this disrupted environment (Figure 4J,J’). These results suggest that Nodal signaling is not required during neurulation for the normal cell behaviors leading to neural tube formation.

**Figure 5:**
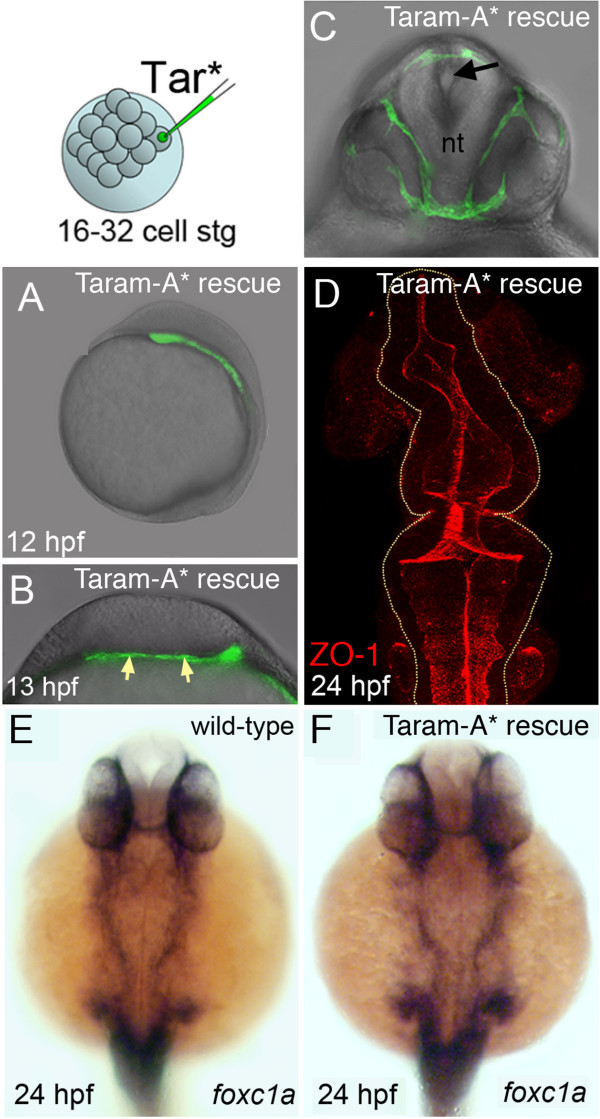
**Mesoderm rescues neural tube morphogenesis in MZ*oep *embryos. (A)** Lateral view of an MZ*oep* embryo previously injected with the activated form of the TGF-β receptor Taram-A* (Tar*) into a single blastomere. Rescued head mesoderm expresses GFP (green). **(B)** Transverse section of Taram-A*-injected embryos show rescued mesoderm (green and arrowed) underlying neural plate at 13 hpf. **(C)** By 24 hpf rescued mesoderm almost surrounds the neural tube (nt) in MZ*oep* embryos. The morphology of the neural tube is rescued and contains a single well-defined midline lumen (black arrow). **(D)** Rescued neural lumen morphology revealed by ZO-1 expression. **(E,F)** Identity, distribution and presence of rescued mesoderm, confirmed by the mesendoderm marker *foxc1a*, in wild-type and Taram-A*-injected MZ*oep* embryos. GFP, green fluorescent protein; hpf, hours post fertilization; MZ*oep*, maternal-zygotic *one-eyed pinhead*; TGF-β, transforming growth factor beta; ZO-1, zonula occludens 1.

To confirm that the neurulation defects result from loss of mesoderm, we rescued the neuroectoderm phenotype by replacing the mesoderm in Nodal-defective embryos. Rescue of mesoderm was achieved by the injection of the constitutively active form of the transforming growth factor beta (TGF-β) receptor Taram-A* [29] into a single peripheral cell at the 16- to 32-cell stage of MZ*oep* embryos (Figure 5). By 12 to 13 hpf Taram-A*-expressing cells populate mesodermal progenitors underneath the neural tissue (Figure 5A,B). By 24 hpf, Taram-A*-expressing cells form part of the cephalic mesoderm of MZ*oep* mutants (Figure 5C) and the normal distribution of mesoderm is rescued (Figure 5E,F). The mutant embryos with rescued mesoderm also now develop a normal organization of the neural tube (Figure 5D). These experiments confirm that mesoderm is able to rescue the neural phenotype of MZ*oep* mutants and strongly suggest that mesoderm is necessary for normal neural tube morphogenesis *in vivo*.

### Movements of the neural plate with and without underlying mesoderm

**Figure 6:**
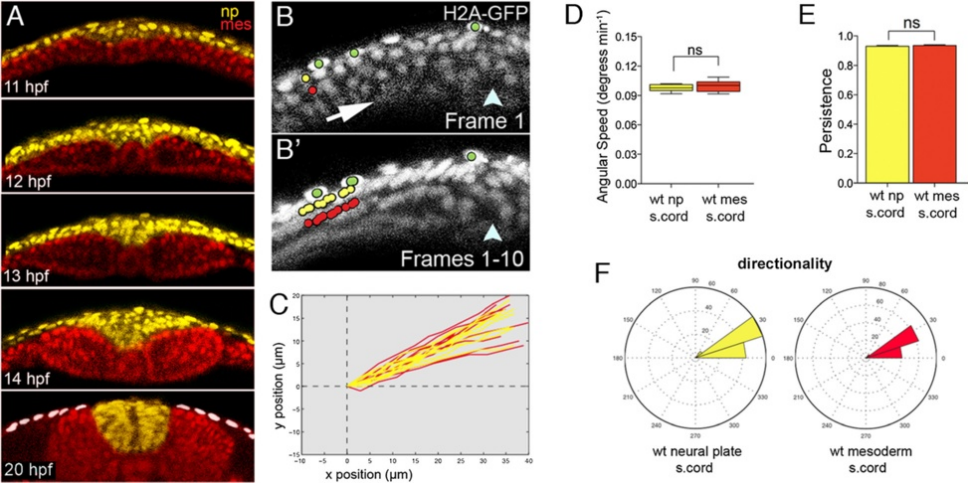
**Neural plate and mesoderm move closely together in early stages of zebrafish neurulation. (A)** Five frames from a time-lapse movie showing relative organization of mesoderm and neural tissue during neurulation. Mesodermal nuclei are pseudocolored red and neural nuclei are pseudocolored yellow. Tissues remain closely apposed throughout convergence and internalization. White dots indicate the enveloping layer. **(B)** Frame 1 from a ten-frame time-lapse sequence at level of spinal cord in a wild-type embryo previously injected with H2B-RFP mRNA (gray). Only the left side of the neural plate is shown. Arrow indicates hypothetical tissue movements and arrowhead indicates position of the embryonic midline. Neural cell nuclei marked with yellow dot. Mesoderm cell nuclei marked with red dot. Enveloping layer cell nuclei marked with green dots. **(B’)** Superimposition of all ten frames from ten-frame time-lapse sequence. The yellow neural nucleus and the red mesodermal nucleus move closely together and in parallel. The green enveloping layer nuclei remain largely immobile. **(C)** Trajectories of neural (yellow) and mesodermal (red) nuclei in wild-type embryos at spinal cord level. **(D)** Analysis of angular speeds show neural plate and adjacent mesoderm move with near identical speeds. **(E)** Analysis of the persistence of movements show neural plate and adjacent mesoderm move with identical persistence. **(F)** Directionality plots for neural plate and adjacent mesoderm cells are nearly identical. H2B-RFP, histone H2B/red fluorescent protein fusion; wt, wild-type.

To investigate the role of the mesoderm during neural plate morphogenesis we first assayed the relative tissue dynamics of the mesoderm and neural plate during wild-type neurulation. We monitored relative cell movements by tracking nuclei using the tg(H2A-GFP) transgenic line [30]. Confocal movies in the transverse plane were made at the level of the prospective anterior spinal cord where the neural plate is composed of one to two layers of cells [24] and the mesoderm cells can be easily identified as they differentiate into somites (Figure 6A and Additional file 1: Movie S1). Between 10.5 and 11.5 hpf, the movements of cells in the neural plate are closely coordinated with cells in the mesoderm as they move towards the midline (Figure 6B,B’,C and Additional file 2: Movie S2). Analysis of angular speed shows that the movements of neural plate and mesoderm cells were tightly coupled during convergence (angular speed: neural plate cells 0.098 degrees/min versus mesodermal cells 0.099 degrees/min, *P* = 0.309 Student’s *t*-test, 200 pairs of cells monitored; Figure 6D). Analysis shows that wild-type cells both in the mesoderm and the neural plate have high persistence towards the dorsal midline (neural plate cells 0.92 versus mesodermal cells 0.93, *P* = 0.450 Student’s *t*-test; Figure 6E). Finally, in wild-type embryos, the neural plate and the mesoderm move with remarkably similar directionality during their dorsal convergence movements (Figure 6F).

**Figure 7:**
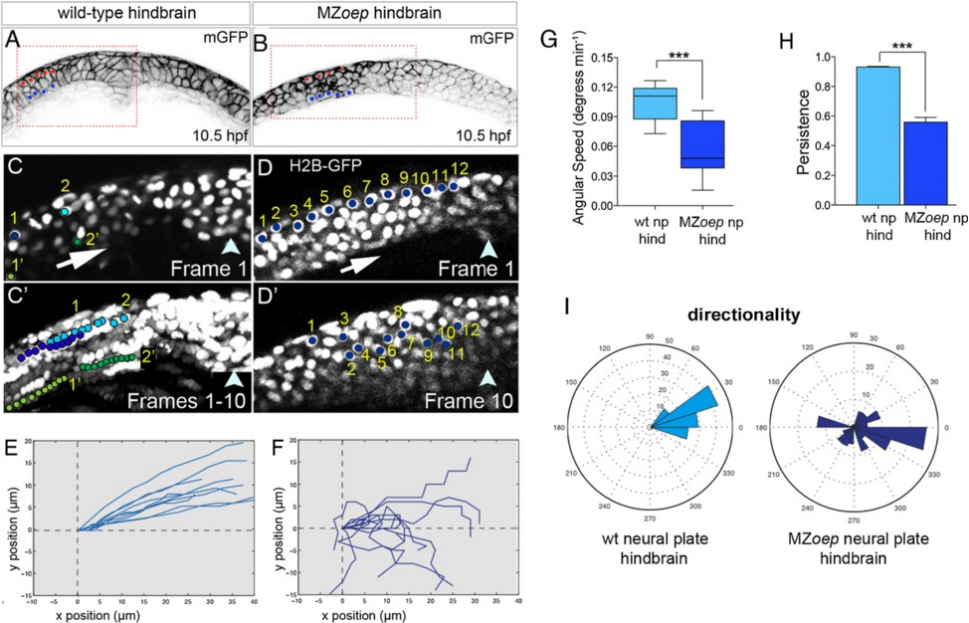
**Disrupted cell movements in MZ*oep *neural plate. (A,B)** Transverse confocal sections of neural plates at hindbrain level in wild-type and MZ*oep* embryo at 10.5 hpf. Cell outlines revealed by membrane-GFP (black). **(C)** Frame 1 from a ten-frame time-lapse sequence at level of wild-type hindbrain. Blue nuclei labeled 1 and 2 are in the superficial layer of the neural plate, and green nuclei labeled 1’ and 2’ are in the deep layer of the neural plate. **(C’)** Superimposition of all ten frames from ten-frame time-lapse sequence. Superficial blue nuclei remain superficial and deep green nuclei remain deep in the converging neural plate. **(D)** Frame 1 from a ten-frame time-lapse sequence at level of hindbrain in MZ*oep* embryo. Twelve nuclei in the superficial layer of the neural plate are marked with blue dots. **(D’)** Position of the 12 nuclei after ten frames shows nuclei are unable to maintain their superficial location in MZ*oep* neural plate. **(E)** Trajectories of neural nuclei in wild-type embryos at hindbrain level. **(F)** Trajectories of neural nuclei at hindbrain level in MZ*oep* embryos. **(G)** Angular speed is significantly reduced in MZ*oep* neural plates (wild-type cells 0.11 degrees/min versus MZ*oep* cells 0.04 degrees/min, ***P <0.0002 Student’s *t*-test). **(H)** Persistence is significantly reduced in MZ*oep* neural plates (wild-type cells 0.92 versus MZ*oep* cells 0.56, ***P <0.0001 Student’s *t*-test). **(I)** Directionality plots for cells in wild-type and MZ*oep* neural plates. GFP, green fluorescent protein; hpf, hours post fertilization; MZ*oep*, maternal-zygotic *one-eyed pinhead*; np, neural plate; wt, wild-type.

We next asked whether the convergence movements of neural plate cells are altered in the absence of mesoderm by making time-lapse movies at early stages of neural plate morphogenesis in MZ*oep* embryos. At 10 hpf the structure of the MZ*oep* neural plate appears normal (Figure 7A,B). However, in comparison to wild-type cells (Figure 7C,C’,E), we found that at 10.5 to 11.5 hpf, MZ*oep* neural plate cell trajectories are very disrupted. MZ*oep* cells cannot maintain their relative superficial/deep positions within the mutant neural plate as they move towards the midline (Figure 7D,D’,F and Additional file 3: Movie S3). In hindbrain regions in the absence of mesoderm, neural plate cells show reduced angular speed compared to wild-type cells (wild-type cells 0.11 degrees/min versus MZ*oep* cells 0.04 degrees/min, *P* <0.0002 Student’s *t*-test; Figure 7G). Neural plate cells in MZ*oep* mutants also show a significant reduction in the persistence of their movements (in hindbrain regions wild-type cells 0.92 versus MZ*oep* cells 0.56, *P* <0.0001 Student’s *t*-test; Figure 7H). In embryos lacking anterior mesoderm, neural plate cells show more random movements and some even display backward displacements (Figure 7I).

**Figure 8:**
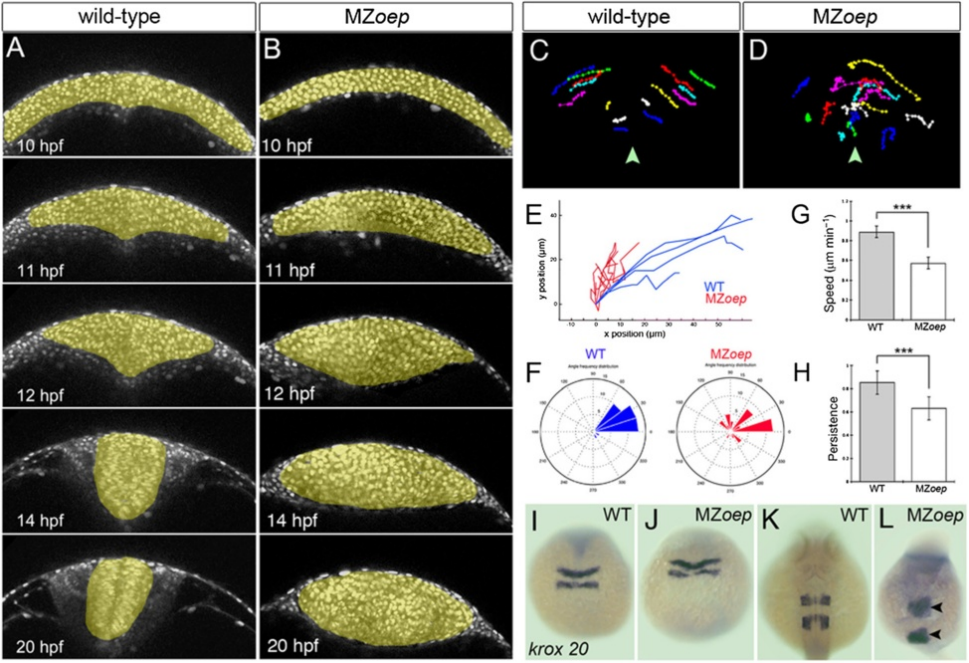
**Neural cell movements in MZ*oep *embryos are highly disrupted through neural keel to neural tube stages. (A,B)** Selected frames from time-lapse sequences showing normal neurulation in a wild-type embryo and disrupted neurulation in a MZ*oep* embryo from 12 hpf. Cells are labeled with H2B-RFP to reveal movements of nuclei (gray). **(C,D)** Tracks of individual neural cell nuclei in wild-type and MZ*oep* embryos at hindbrain level. Midline is marked with arrowhead. **(E)** Trajectory plots of cells in wild-type and MZ*oep* hindbrain primordia. **(F)** Directionality plots for cells in wild-type and MZ*oep* neural plates. **(G,H)** Speed and persistence of neural cell movements are disrupted from 12 hpf onwards in MZ*oep* embryos (linear speed μm/min: wild-type cells 0.87 versus MZ*oep* cells 0.56, ***P <0.0001 Student’s *t*-test; persistence: wild-type cells 0.85 versus MZ*oep* cells 0.63, ***P <0.0002 Student’s *t*-test). **(I,J,K,L)** The hindbrain marker *krox20* shows that the anterior-posterior pattern in hindbrain region is only mildly disrupted despite abnormal cell movements in MZ*oep* neural primordia. H2B-RFP, histone H2B/red fluorescent protein fusion; hpf, hours post fertilization; MZ*oep*, maternal-zygotic *one-eyed pinhead*; wt, wild-type.

The disrupted movements of neural plate cells in MZ*oep* embryos result in an abnormally shaped neural primordium by 12 hpf (Figure 8A,B). Time-lapse analyses from this point onwards reveal increasingly disrupted morphogenesis of the MZ*oep* neural primordium (Additional file 4: Movie S4). MZ*oep* cells fail to internalize normally and fail to form a normal neural keel. While in wild-type embryos neural cells move smoothly towards the midline during the neural keel formation, MZ*oep* neural cells show more chaotic trajectories (Figure 8C,D,E,F). In addition, analysis of single cells show that MZ*oep* cells are often able to move into the contralateral sides of the developing rod (Figure 8D), a behavior almost never seen in the normal neural rod unless driven by a midline division [14]. Cell speed and persistence are both reduced in MZ*oep* from 12 hpf onwards (Figure 8G,H). Despite these extensive alterations in tissue movements, the general organization of hindbrain segmentation appears to be largely maintained through this period of development (Figure 8I,J,K,L), suggesting little mixing of cells occurs along the anterior-posterior axis.

### The development of polarity in MZ*oep* neural plate

**Figure 9:**
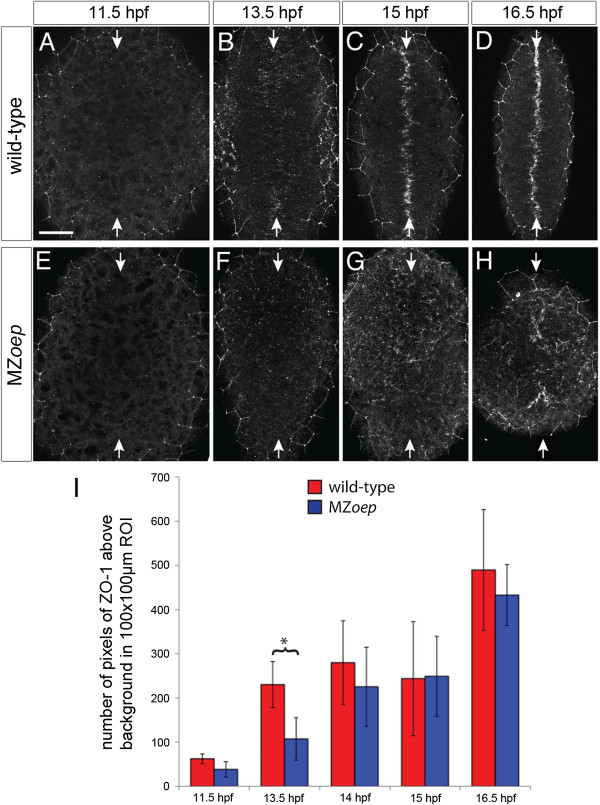
**Apical polarity development in MZ*oep*.** ZO-1 staining in **(A,B,C,D)** wild-type and **(E,F,G,H)** MZ*oep* embryos at different stages of neurulation. All images are maximum confocal projections of six consecutive z-levels taken from a dorsal view at posterior hindbrain and anterior spinal cord regions. Anterior is up. White arrows indicate the dorsal midline of the embryo. The ZO-1 staining surrounding the edge of the neural tissue is from the polarized enveloping layer overlying the neural tissue. Scale bar is 50 μm. **(I)** The average number of pixels of ZO-1 staining over time for MZ*oep* and wild-type embryos. At 13.5 hpf the number of ZO-1 puncta in MZ*oep* embryos is significantly different to wild-type (*P <0.01, two-way ANOVA), indicating that ZO-1 polarization is delayed by 30 minutes in MZ*oep* embryos. At subsequent time points (14, 15 and 16 hpf), there is no significant difference between the wild-type and MZ*oep* embryos. Error bars indicate the standard deviation. ANOVA, analysis of variance; hpf, hours post fertilization; MZ*oep*, maternal-zygotic *one-eyed pinhead*; ZO-1, zonula occludens 1.

Although our analysis of the neural primordium at 24 hpf shows several aspects of the polarized neuroepithelium are intact in MZ*oep* (Figure 1I,K,L), it is possible that the development of apicobasal polarity in the neural cells is disrupted at earlier time-points. To address this we assessed the onset of ZO-1 expression as this should be an assay of when cells begin to make apical cell-cell junctions. At 11.5 hpf there is almost no ZO-1 immunoreactivity in either wild-type or MZ*oep* neural plate (Figure 9A,E). By 13.5 hpf the number of ZO-1 puncta has increased in both wild-type and MZ*oep* neural plate, but is more significantly elevated in wild-type (*P* <0.01, two-way analysis of variance (ANOVA); Figure 9B,F). From 14 hpf the number of ZO-1 puncta is not significantly different between wild-type and MZ*oep*; however, in the wild-type ZO-1 is found concentrated close to the neural midline, while in MZ*oep* the ZO-1 puncta are more widely scattered (Figure 9C,D,G,H,I). These results suggest the accumulation of ZO-1 into distinct puncta is transiently delayed by 30 minutes in the MZ*oep* mutant neural plate.

## Discussion

During zebrafish neurulation neural plate cells first converge towards the dorsal midline, then internalize to form the neural keel, which subsequently transforms via a solid rod primordium to become the neural tube. In this work we show that underlying mesoderm is required for the coordinated and persistent movements of the neural plate cells during convergence and keel formation.

The loss of mesoderm has dramatic effects on the directionality and coordination of cell movements within the neural plate. Although the initial structure of the neural plate at 10 hpf appears normal, cells are unable to converge normally towards the midline and generate a neural keel. Despite moving in the general direction of the midline, cell velocity is reduced and they are unable to maintain persistent directionality. In comparison to wild-type cells, the nuclei in the neural plates of mesodermless embryos are unable to maintain their relative positions within the depth of the neural plate. We do not know the mechanism by which mesoderm influences the neural plate cells. One possibility is the presence of the underlying mesoderm provides a physical barrier to constrain the space in which neural plate cells can move, that is, it forms the floor of a thin corridor that could restrict the movement of the neural plate cells to the mediolateral axis. Alternatively there may be an active coupling of the mesoderm and the neural plate such that neural plate convergence is at least partially driven by the convergence movements of the mesoderm. Our time-lapse observations show that the movements of neural plate cells and mesoderm cells are indeed very closely coupled during convergence towards the midline (Figure 6B). Although they are in close proximity, there is probably no physical contact between cells of the neural plate and mesoderm because there is an intervening basal lamina. The basal lamina will be enriched with extracellular matrix (ECM) proteins such as laminin and fibronectin and this raises the possibility that they might have a potential role in coordinating the movements of mesoderm and neural plate. ECM has been proposed to play a central role in a variety of processes that could be relevant to this interaction, including cell movements and tissue rearrangements [31-34]. In frogs, initial gastrulation movements are marked by the involution and subsequent migration of the mesoderm under the ectodermal blastocoel roof towards the animal pole [35]. A number of studies have shown that mesoderm migration depends on a well-developed fibronectin matrix, which is deposited by the overlying blastocoel roof [36-40]. Interestingly, blocking experiments against fibronectin-fibronectin assembly results in loss of tissue apposition between mesoderm and blastocoel roof and altered tissue dynamics during blastopore closure [40]. In the future it will be important to address whether ECM components contribute to neural plate movements and to the coupling of mesoderm to neural plate in the fish. The role of the ECM could be quite complex as the ECM itself may well be remodeled during this phase of morphogenesis [38,39] and indeed the ECM may move along with the moving cells and tissues [41,42].

Since the development of cell polarity is likely to be a critical factor in neural tube formation in the zebrafish [10,13,14,17,18] we asked whether loss of mesoderm had a major influence on the development of polarity in the neural cells. One measure of polarity in this developing epithelium would be the development of cell-cell junctions. Thus, using ZO-1 immunoreactivity as an assay of polarity we found its expression is initially detected in the neural plate at approximately the same time in MZ*oep* and wild-type cells but that its accumulation into distinct puncta is delayed by approximately 30 minutes in MZ*oep*. After this short delay polarization continues with the same schedule as wild-type, although the distribution of ZO-1 puncta is more scattered in the MZ*oep* tissue. It is possible that the 30-minute delay represents a genuine delay in the cellular mechanisms that drive polarization, but it may also be possible that the more random movements of the MZ*oep* cells will lead to cell-cell contacts between neighboring cells becoming more transient and this could destabilize distinct cell-cell junctions. Since we have previously shown by ectopic transplantation strategies that the dorsal mesoderm is not required for neural polarization and lumen formation [18], we favor the view that mesoderm is more important in directing neural cell movements than initiating neural cell polarization. It is possible that mesoderm acts to coordinate the orientation of neural polarity and that this could influence how the neural cells move.

One further possibility is that the defective cell movements in MZ*oep* result not from a lack of mesoderm but rather from an unfavorable interaction of the neural plate with the underlying yolk, which is a tissue that it would not normally be exposed to. This possibility suggests that loss of mesoderm simply allows the neural plate to interact with the yolk rather than removing a positive influence of mesoderm. While we cannot rule out this possibility, we feel the tightly coupled movements of mesoderm and neural plate that our analysis reveals, plus the previously published work suggesting mesoderm is required for neural plate movements in *Xenopus* explants [7,8], all point to a positive influence of mesoderm on the neural plate.

At present the motive forces driving the neural plate movements of convergence and internalization in the fish are almost completely unknown. In addition to potential influences for the mesoderm, other non-neural tissues could also be involved (reviewed by Gordon [43] and Colas and Schoenwolf [44]). In chicken embryos for instance, *in vitro* explant experiments suggest that the adjacent non-neural ectoderm provides a ‘pushing force’ required to shape neural fold formation and encourage dorsal closure of the neural tube [45,46]. More recently, functional studies and live tissue analyses in amphibian embryos indicate that pulling forces generated by the deep layer of non-neural ectoderm are required to complete neural tube closure in *Xenopus* and this partially depends on the cell adhesion molecule E-cadherin and the ECM receptor integrin-β1 [47].

Finally our observations show that the location and orientation of cell divisions that usually take place across the midline of the neural rod are severely disrupted by loss of mesoderm. The regulation of these morphogenetically powerful divisions [13,14] has recently been shown to be under the control of the non-canonical Wnt receptor Frizzled 7 [15] and the polarity protein Scribble [16]. Although these divisions are disrupted in the absence of mesoderm, this is not the primary cause of the neural tube defects in these embryos because, unlike the other morphogenetic mutants [13-16], inhibiting these divisions in the mesodermless embryos does not rescue neural tube morphogenesis. The primary cause of the mesodermless neural phenotype is thus established before and is independent of the midline divisions. In contrast, apart from misregulation of the oriented divisions, in the *vangl2*, *fz7* and *scrib* mutants any other cellular or molecular defects related to neural tube development must be relatively minor because their neural tube defects are lost when these divisions are blocked [13-16].

## Conclusions

We show that the movements of cells in the zebrafish neural plate are highly coordinated with the movements of the underlying mesoderm during the convergence and internalization movements of neurulation. Our analyses of mesodermless embryos demonstrate that the underlying mesoderm is required for the coordinated convergence movements of the zebrafish neural plate cells *in vivo*.

## Methods

All procedures were approved by the College Research Ethics Committee at King’s College London (London, UK) and covered by the Home Office Animals (Scientific Procedures) Act 1986 (ASPA) project licence.

### Zebrafish strains

Zebrafish embryos were collected and staged using standard protocols [48] and provided by the King’s College London fish facility. The following zebrafish alleles were used: wild-type TL, *oeptz*^*257*^[20], *cyc*^*m294*^[26], *ntl*^*160*^[49], *syut*^*4*^[50] and tg(HUC-GFP) [51]. MZ*oep* mutant embryos were generated by crossing *oeptz*^*257*^ adult zebrafish previously rescued by injection of *oep* mRNA at one-cell stage [21]. For time-lapse confocal movies tg(H2A-GFP) [30] was used. Embryos were grown at 28.5°C and staged according to morphology [52] and age hpf. mRNA and morpholino injections PCS2+ vectors carrying cDNA fragment encoding for Pard3-GFP (kind gift of Dr Alexander Reugels, University of Cologne, Cologne, Germany), membrane-GFP [53], membrane-RFP [54] and histone H2B/red fluorescent protein fusion (H2B-RFP) [55] were used in this study. Capped RNAs were transcribed using SP6 and T7 RNA polymerase using the mMESSAGE mMACHINE Kit (Ambion, Austin, TX, USA) and 180 pg Pard3-GFP, 120 pg of membrane-GFP, 150 pg of membrane-RFP and 150 pg of H2B-RFP mRNA were injected into wild-type or MZ*oep* embryos. For ubiquitous expression, mRNA was injected at the one-cell stage. For rescue experiments, MZ*oep* embryos were injected with 8 to 10 pg of Taram-A* alone or co-injected with mGFP mRNA in one of the marginal blastomeres at 16- or 32-cell stage [29]. Morpholino oligonucleotides (MOs; Gene Tools, Philomath, OR, USA) were dissolved in water to a concentration of 4 mM and stored at −20°C. All MOs were injected at one-cell stage. To generate MZ*oep*/*spt* double-mutant embryos, a combination of two previously characterized MOs [56] against the *spt* gene were injected into MZ*oep* embryos: *spt1*-MO 5′-AGCCTGCATTATTTAGCCTTCTCTA-3′ (0.4 pmoles/embryo) and *spt2*-MO 5′-GATGTCCTCTAAAAGAAAATGTCAG-3′ (0.4 pmoles/embryo). These concentrations of morpholinos have previously been shown to mimic the phenotype of the *spadetail* mutant [56].

### *In situ* hybridization

Antisense RNA probes were synthesized with digoxigenin RNA labelling kit (Roche, Basel, Switzerland) using plasmid-containing cDNA for *left1*[27], *pitx2*[28], *krox*-*20*[57] and *foxc1a*[58]. Embryos were fixed and stained at appropriate stages. To confirm that neural tube morphology was rescued when Taram-A*-expressing cells became mesoderm, we assessed the expression of the cephalic mesoderm marker, *foxc1a*[58].

### Antibody staining

For whole mount and cryosection immunostaining, embryos were fixed in 4% paraformaldehyde (PFA) at 4°C overnight at different stages (between 11 to 24 hpf). Embryos were blocked in 10% normal goat serum (Sigma-Aldrich, St Louis, MO, USA) for 2 hours at room temperature. The following primary antibodies were used in this study: mouse-anti-ZO-1 (339111; Zymed Laboratories, South San Francisco, CA, USA) at 1:300; rabbit-anti-aPKC (C-20; Santa Cruz Biotechnology, Dallas, TX, USA) at 1:500; rabbit-anti-GFAP (Z0334; DakoCytomation, Glostrup, Denmark) at 1:500; mouse-anti-MF-20 (Developmental Studies Hybridoma Bank, Iowa City, IA, USA) at 1:50; mouse-anti-acetylated-tubulin (T6793; Sigma-Aldrich); and rabbit-anti-phospho-histone H3 (Upstate Biotechnology, Lake Placid, NY, USA) at 1:200, diluted in 2.5% normal goat serum (Sigma-Aldrich). For secondary antibodies, anti-rabbit and anti-mouse Alexa 488, Alexa 568 and Alexa 633 (Molecular Probes, Eugene, OR, USA) were used at 1:800 in 2.5% normal goat serum. Sections were cut every 14 to 16 μm on a Leica 5100 or Cryo-Star HM 560 MV Micron microtomes.

### SB-431542 treatment

To block Nodal signaling, wild-type embryos were treated with SB-431542 inhibitor [22]. SB-431542 (4-(4-(1,3-benzodioxol-5-yl)-5-(2-pyrindinyl)-1H-imidazol-2-yl)benzamide; Tocris Bioscience, Bristol, UK) was dissolved in dimethyl sulfoxide (DMSO) to make a 100 mM stock and stored at −20°C. For drug treatment, approximately 30 embryos at two- to four- cell stage or before neurula stage (70% epiboly, 7 to 8 hpf) were put in a small dish containing 100 μM of SB-431542 dissolved in embryo medium and raised at 28.5°C.

### Cell division inhibition

To inhibit cell division, wild-type and MZ*oep* embryos were treated with a combination of 150 μM aphidicolin (Biomol, Hamburg, Germany) and 50 mM hydroxyurea (Sigma-Aldrich) dissolved in 4% DMSO. Inhibition of cell division was performed at tail-bud stages and quantification of PH3+ cells was done for equivalent tissue volumes (360 μm in length × 130 μm in width × 90 μm in depth).

### Cell transplantation

Wild-type and MZ*oep* mutant embryos were used as host and donor embryos. To identify donor cells from host cells, donor embryos were previously injected with either membrane-GFP or membrane-RFP. Between sphere and dome stage (4.0 to 4.3 hpf), dechorionated embryos were transferred into an agarose chamber and about 30 donor cells were transplanted into the prospective hindbrain region of a host embryo.

### Time-lapse imaging

For time-lapse confocal analysis, embryos were manually dechorionated at tail-bud stage (10 to 11 hpf) and mounted in 1.2% low melting point agarose (Sigma-Aldrich) in embryo medium (E3). For neural plate mesoderm tracking analysis, embryos were imaged in transverse view at the level of the first or second somites. Confocal images were taken 5 to 8 μm apart, using a 40× long working distance water immersion objective in an environment chamber at 28.5°C. Z-stacks were collected at 5-minute intervals, usually starting between 10 to 11 hpf and continuing through to 18 to 20 hpf.

### Cell movement analysis

ImageJ software was used to assemble the image stacks into time-lapse movies. Cropped time-lapse movies were then assembled in QuickTime Pro (Apple, Cupertino, CA, USA) and Adobe Photoshop CS4 (Adobe Systems, San Jose, CA, USA) was used for final figure assembly. For nuclei tracking analysis, tg(H2A-GFP) transgenic zebrafish [30], or embryos expressing H2B-RFP, membrane-GFP, Pard3-GFP or membrane-RFP mRNA were used. For tracking, neuroectodermal and mesodermal cells were identified according to their position in their respective germ layer and in more ambiguous cases, time lapses were played back in order to recognize in which layer a cell was located. Usually, ten consecutive frames for each cell were analyzed using ‘Manual Tracking’ plugins for ImageJ (http://rsb.info.nih.gov/ij/plugins/track/track.html). The persistence of a cell was defined by the ratio between the linear distance towards the midline and total migration over total path tracks. Angular cell movements during dorsal migration were calculated according to previous measurements. To calculate the angular speed of a travelling cell (degrees/min), the distance between its start and end points was first determined. This distance was considered to be a chord of the respective germ layer, whose curvature was projected to produce a circle. Then, the diameter of the projected circle was calculated and the angle θ subtended by chord was calculated using:

$$\theta =2*\arcsin\left(\frac{\mathrm{chd\theta}}{2r}\right)$$

where chdθ equals the length traveled by a cell (the chord) and 2r equals the diameter of the projected circle. For each experimental condition, six to ten embryos from independent experiments were analyzed. To test for significance between mean values, Student’s *t*-test was applied between wild-type and MZ*oep* embryos. A probability of 0.05 or less was accepted as statistically significant. For each condition, the standard error of the mean (SEM) was calculated. Analysis and graphical representations were performed using GraphPad Prism (GraphPad Software, La Jolla, CA, USA) and MATLAB (R14b; MathWorks, Cambridge, UK) statistical programs.

### Quantifying the appearance of ZO-1 puncta

For analysis of the appearance of ZO-1 puncta in wild-type and MZ*oep* embryos, the following steps were taken to ensure that staining and imaging across specimens was as consistent as possible. 1) Immunohistochemistry: at the time of fixation, wild-type and MZ*oep* embryos were staged under the dissecting microscope according to the number of somites. Embryos were fixed in 4% PFA from the same aliquot and fixed overnight at 4°C for the same time period. Embryos were then washed in fresh PBS, dehydrated into methanol and put at −20°C to permeabilize the embryos. When all embryos of all stages had been placed at −20°C for at least 1 hour, the normal immunohistochemistry protocol was followed with all tubes of embryos being treated identically. For all stages, the same aliquot of antibody was used at the same dilution. 2) Imaging: after washing of the secondary antibody, all embryos were mounted for dorsal view imaging through the hindbrain. Confocal setting including laser power, gain, offset, pinhole and averaging were set at the beginning of imaging and left unchanged throughout. All embryos were imaged on the same day and the same z-stack parameters were used for each embryo (25 z-slices at 3 μm intervals), starting from the dorsal most part of the neural primordium. 3) Data analysis: a histogram of all pixel intensities (256 gray levels) was derived from a large region of interest (ROI) (100 μm × 100 μm) in each of three different z-levels per specimen. z-levels at comparable dorsoventral depths were chosen for the analysis across embryos to minimize the change in intensity that occurs with z-depth. Care was taken to avoid sampling any signal from the polarized enveloping layer and from the ventral most z-levels because in wild-type embryos these polarize earlier than the rest of the tissue and are not present in MZ*oep* mutants due to the loss of ventral midline-specified cells [21]. For each time point, two to eight mutant or wild-type embryos were analyzed. The average maximum background pixel intensity was calculated from maximum intensity measurements at five locations in each chosen z-slice. Background pixel intensity was sampled from a smaller ROI (10 μm × 10 μm), avoiding obvious real signal. Pixels below this cut-off value were regarded as background and any pixels above were counted as signal. Finally, as the sampled number of pixels was the same for every measurement, the average number of pixels above background was calculated for wild-type or MZ*oep* embryos at each stage.

## Abbreviations

Ac-tub: Anti-acetylated tubulin antibody; ANOVA: Analysis of variance; ASPA: Animals (Scientific Procedures) Act 1986; aPKC: Atypical protein kinase C; cas: *casanova*; CDI: Cell division inhibitor; cyc: *cyclops*; DMSO: Dimethyl sulfoxide; ECM: Extracellular matrix; GFAP: Glial fibrillary acidic protein; H2B-RFP: Histone H2B/red fluorescent protein fusion; hpf: Hours post fertilization; MO: Morpholino oligonucleotide; MZoep: Maternal-zygotic *one-eyed pinhead*; OV: Otic vesicle; Pard3-GFP: Green fluorescent protein/polarity protein partitioning defective 3 fusion; PBS: Phosphate-buffered saline; PCP: Planar cell polarity; PFA: Paraformaldehyde; PH3: Phospho-histone 3 marker; ROI: Region of interest; SEM: Standard error of the mean; TGF-β: Transforming growth factor beta; wt: wild-type; ZO-1: Zonula occludens 1.

## Competing interests

The authors declare that they have no competing interests.

## Authors’ contributions

CA conceived and designed the study, undertook data collection and analysis and wrote the manuscript. MT undertook data collection and analysis. GG undertook data collection and analysis and wrote the manuscript. MC undertook data collection and analysis. CCF undertook data collection and analysis. JC conceived and designed the study, arranged financial support and wrote the manuscript. All authors read and approved the final manuscript.

## Supplementary Material

Additional file 1: Movie S1 Time-lapse movie taken in the transverse plane showing both left- and right-hand sides of the neural plate and underlying mesoderm at the level of anterior spinal cord. Nuclei are expressing H2A-GFP. Movie starts at neural plate stage and ends at neural keel stage. In the last frame the neural keel is outlined by yellow dots and the mesoderm forming the somites and notochord is outlined by red dots. The rectangle seen in the first frame outlines the approximate tissue area shown in Movies S2 and S3. Click here for file

Additional file 2: Movie S2 Time-lapse movie taken in the transverse plane through the left-hand side of the neural plate and underlying mesoderm at the level of anterior spinal cord (approximate area of rectangle in first frame of Movie S1). The midline of this wild-type embryo is indicated with an arrow. The nuclei of neural plate cells (yellow dots) and subjacent mesoderm cells (red dots) are seen to move in a closely coordinated way. Nuclei of the overlying enveloping layer (blue dots) are seen to remain stationary. Click here for file

Additional file 3: Movie S3 Time-lapse movie taken in the transverse plane through the left-hand side of the neural plate at the level of the hindbrain (approximate area of rectangle in first frame of Movie S1). The midline of this MZ*oep* embryo is indicated with an arrow. In contrast to normal embryos, 12 nuclei (blue dots) that initially lie at the superficial surface of the neural plate are seen to distribute themselves throughout the deeper layers of the neural plate in subsequent frames. Nuclei that appear only in later frames at the superficial surface are marked with red and green dots. The depth of the neural plate is also seen to enlarge as the movie progresses. Click here for file

Additional file 4: Movie S4 Single confocal plane comparing neurulation movements between a wild-type embryo (wt) and an MZ*oep* mutant at level of the hindbrain. Cells are labelled with membrane-GFP (m:GFP) and nuclear-RED (n:RED). Otic vesicle (OV), at the end of the movie, is used as a landmark for hindbrain. The time between frames is 5 minutes, duration 8.3 hours (between 12 to 20 hpf). Arrows indicate the position of the midline. Click here for file

## References

[B1] Ueno N Greene ND Planar cell polarity genes and neural tube closure Birth Defects Res 2003 69 318 324 10.1002/bdrc.10029 14745972

[B2] Holtfreter J Die totale exogastrulation, eine selbstablösung des ektoderms vom entomesoderm Roux' Arch F Entw Mech 1933 129 669 6793 10.1007/BF00656583 28353925

[B3] Takaya H On the types of neural tissue developed in connection with mesodermal tissues Ann Zool Jap 1956 4 287 292

[B4] Chen ZF Behringer RR Twist is required in head mesenchyme for cranial neural tube morphogenesis Genes Dev 1995 9 686 699 10.1101/gad.9.6.686 7729687

[B5] Zhao Q Behringer RR de Crombrugghe B Prenatal folic acid treatment suppresses acrania and meroanencephaly in mice mutant for the Cart1 homeobox gene Nat Genet 1996 13 275 283 10.1038/ng0796-275 8673125

[B6] Copp AJ Greene ND Murdoch JN The genetic basis of mammalian neurulation Nat Rev Genet 2003 4 784 793 10.1038/nrg1181 13679871

[B7] Poznanski A Minsuk S Stathopoulos D Keller R Epithelial cell wedging and neural trough formation are induced planarly in *Xenopus*, without persistent vertical interactions with mesoderm Dev Biol 1997 189 256 269 10.1006/dbio.1997.8678 9299118

[B8] Elul T Keller R Monopolar protrusive activity: a new morphogenetic cell behaviour in the neural plate dependent on the vertical interactions with the mesoderm in *Xenopus* Dev Biol 2000 224 3 19 10.1006/dbio.2000.9746 10898957

[B9] Jessen JR Topczewski J Bingham S Sepich DS Marlow F Chandrasekhar A Solnica-Krezel L Zebrafish trilobite identifies new roles for Strabismus in gastrulation and neuronal movements Nat Cell Biol 2002 8 610 615 12105418 10.1038/ncb828 PMC2219916

[B10] Hong E Brewster R N-cadherin is required for the polarized cell behaviors that drive neurulation in the zebrafish Development 2006 133 3895 3905 10.1242/dev.02560 16943271

[B11] Lowery LA Sive H Strategies of vertebrate neurulation and a reevaluation of teleost neural tube formation Development 2004 2057–2067 132 10.1016/j.mod.2004.04.022 15327780

[B12] Clarke J Role of polarized cell division in zebrafish neural tube formation Curr Opin Neurobiol 2009 19 134 138 10.1016/j.conb.2009.04.010 19447605 PMC2791883

[B13] Ciruna B Jenny A Lee D Mlodzik M Schier AF Planar cell polarity signalling couples cell division and morphogenesis during neurulation Nature 2006 439 220 224 10.1038/nature04375 16407953 PMC1417047

[B14] Tawk M Araya C Lyons DA Reugels AM Girdler GC Bayley PR Hyde DR Tada M Clarke JD A mirror-symmetric cell division that orchestrates neuroepithelial morphogenesis Nature 2007 446 797 800 10.1038/nature05722 17392791

[B15] Quesada-Hernandez E Caneparo L Schneider S Winkler S Liebling M Fraser SE Heisenberg CP Stereotypical cell division orientation controls neural rod midline formation in zebrafish Curr Biol 1966–1972 2010 20 10.1016/j.cub.2010.10.009 20970340

[B16] Zigman M Trinh le A Fraser SE Moens CB Zebrafish neural tube morphogenesis requires Scribble-dependent oriented cell divisions Curr Biol 2011 21 79 86 10.1016/j.cub.2010.12.005 21185191 PMC4308621

[B17] Buckley CE Ren X Ward LC Girdler GC Araya C Green MJ Clark BS Link BA Clarke JDW Mirror-symmetric microtubule assembly and cell interactions drive lumen formation in the zebrafish neural rod EMBO J 2013 32 30 44 23202854 10.1038/emboj.2012.305 PMC3545300

[B18] Girdler CG Araya C Ren X Clarke JD Developmental time rather than local environment regulates the schedule of epithelial polarization in the zebrafish neural rod Neural Development 2013 8 5 10.1186/1749-8104-8-5 23521850 PMC3623869

[B19] Aquilina-Beck A Llagan K Liu Q Liang JO Nodal signalling is required for closure of the anterior neural tube in zebrafish BMC Dev Biol 2007 7 126 146 10.1186/1471-213X-7-126 17996054 PMC2214732

[B20] Schier AF Neuhauss SC Helde KA Talbot WS Driever W The one-eyed pinhead gene functions in mesoderm and endoderm formation in zebrafish and interacts with no tail Development 1997 124 327 342 9053309 10.1242/dev.124.2.327

[B21] Gritsman K Zhang J Cheng S Heckescher E Talbot WS Schier AF The EGF-CFC protein one eye-pinhead is essential for nodal signaling Cell 1999 97 121 132 10.1016/S0092-8674(00)80720-5 10199408

[B22] Imman GJ Nicolas FJ Callahan JF Harling JD Gaster LM Reith AD Laping NJ Hill CS SB-431542 is a potent and specific inhibitor of transforming growth factor-beta superfamily type I activin receptor-like kinase (ALK) receptors ALK4, ALK5, and ALK7 Mol Pharmacol 2002 62 65 74 10.1124/mol.62.1.65 12065756

[B23] Kimmel CB Warga RM Kane DA Cell cycles and clonal strings during formation of the zebrafish central nervous system Development 1994 120 265 276 8149908 10.1242/dev.120.2.265

[B24] Geldmacher-Voss B Reugels AM Pauls S Campos-Ortega JA A 90-degree rotation of the mitotic spindle changes the orientation of mitoses of zebrafish neuroepithelial cells Development 2003 130 3767 3780 10.1242/dev.00603 12835393

[B25] Griffin KJ Kimelman D One-Eyed Pinhead and Spadetail are essential for heart and somite formation Nat Cell Biol 2002 4 821 825 10.1038/ncb862 12360294

[B26] Hatta K Kimmel CB Ho RK Walker C The cyclops mutation blocks specification of the floor plate of the zebrafish central nervous system Nature 1991 350 339 341 10.1038/350339a0 2008211

[B27] Thisse C Thisse B Antivin, a novel and divergent member of the TGFbeta superfamily regulates anteroposterior endoderm patterning in zebrafish Mech Dev 1999 126 229 240 10.1242/dev.126.2.229 9847237

[B28] Essner JJ Brandford WW Zhang J Yost HJ Mesendoderm and left-right brain, heart and gut development are differentially regulated by pitx2 isoforms Development 2000 127 1081 1093 10662647 10.1242/dev.127.5.1081

[B29] Renucci A Lemarchandel V Rosa F An activated form of type I serine/threonine kinase receptor TARAM-A reveals a specific signalling pathway involved in fish head organiser formation Development 1996 122 3735 3743 9012495 10.1242/dev.122.12.3735

[B30] Pauls S Geldmacher-Voss B Campos-Ortega JA A zebrafish histone variant H2A.F/Z and transgenic H2A.F/Z:GFP fusion protein for in vivo studies of embryonic development Dev Genes Evol 2001 211 603 610 10.1007/s00427-001-0196-x 11819118

[B31] Miner JH Yurchenco PD Laminin functions in tissue morphogenesis Annu Rev Cell Biol 2004 20 255 284 10.1146/annurev.cellbio.20.010403.094555 15473841

[B32] Rozario T DeSimone DW The extracellular matrix in developmental and morphogenesis: a dynamic view Dev Biol 2010 341 126 140 10.1016/j.ydbio.2009.10.026 19854168 PMC2854274

[B33] Zamir EA Rongish BJ Little CD The ECM moves during primitive streak formation-computational of ECM versus cellular motion PLoS Biol 2008 6 e247 10.1371/journal.pbio.0060247 18922043 PMC2567004

[B34] Winklbauer R Mesodermal cell migration during *Xenopus* gastrulation Dev Biol 1990 142 155 168 10.1016/0012-1606(90)90159-G 2227092

[B35] Boucaut JC Darribere T Fibronectin in early amphibian embryos. Migrating mesodermal cells contact fibronectin established prior to gastrulation Cell Tissue Res 1983 234 135 145 6640612 10.1007/BF00217407

[B36] Winklbauer R Nagel M Selchow A Wacker S Mesoderm migration in the *Xenopus* gastrula Int J Dev Biol 1996 40 305 311 8735942

[B37] Longo D Percie SM Skalak TC Davidson L Marsden M Dzamba B DeSimone DW Multicellular computer simulation of morphogenesis: blastocoel roof thinning and matrix assembly in *Xenopus laevis* Dev Biol 2004 271 210 222 10.1016/j.ydbio.2004.03.021 15196962

[B38] Davidson LA Keller R DeSimone DW Assembly and remodeling of the fibrillar fibronectin extracellular matrix during gastrulation and neurulation in *Xenopus laevis* Dev Biol 2004 231 888 895 10.1002/dvdy.20217 15517579

[B39] Davidson LA Dzamba BD Keller R DeSimone DW Live imaging of cell protrusive activity, extracellular matrix assembly and remodeling during morphogenesis in the frog, *Xenopus laevis* Dev Dyn 2008 237 2684 2692 10.1002/dvdy.21600 18629871 PMC2628587

[B40] Rozario T Dzamba B Weber GF Davidson LA DeSimone DW The physical state of fibronectin matrix differentially regulates morphogenetic movements *in vivo* Dev Biol 2009 327 386 98 10.1016/j.ydbio.2008.12.025 19138684 PMC2829434

[B41] Czirók A Rongish BJ Little CD Extracellular matrix dynamics during vertebrate axis formation Dev Biol 2004 268 111 122 10.1016/j.ydbio.2003.09.040 15031109

[B42] Zamir EA Czirók A Cui C Little CD Rongish BJ Mesodermal cell displacements during avian gastrulation are due to both individual cell-autonomous and convective tissue movements Proc Natl Acad Sci U S A 2006 103 19806 19811 10.1073/pnas.0606100103 17179040 PMC1705812

[B43] Gordon R A review of the theories of vertebrate neurulation and their relationship to the mechanics of the neural tube birth defects J Embryol Exp Morphol 1985 89 229 355 3913733

[B44] Colas JF Schoenwolf GC Towards a cellular and molecular understanding of neurulation Dev Dyn 2001 221 117 145 10.1002/dvdy.1144 11376482

[B45] Alvarez IS Schoenwolf GC Expansion of surface epithelium provides the major extrinsic force for bending of the neural plate J Exp Zool 1992 261 340 348 10.1002/jez.1402610313 1629665

[B46] Hackett DA Smith JL Schoenwolf GC Epidermal ectoderm is required for full elevation and convergence during bending of the avian neural plate Dev Dyn 1997 210 397 406 10.1002/(SICI)1097-0177(199712)210:4<397::AID-AJA4>3.0.CO;2-B 9415425

[B47] Morita H Kajiura-Kobayashi H Takagi C Yamamoto TS Nonaka S Ueno N Cell movements of the deep layer of non-neural ectoderm underlie complete neural tube closure in *Xenopus* Development 2012 139 1417 1426 10.1242/dev.073239 22378637

[B48] Westerfield M The Zebrafish Book. A Guide for the Laboratory Use of Zebrafish (Danio rerio). 4th edition 2000 Eugene, OR: University of Oregon Press

[B49] Schulte-Merker S van Eeden FJ Halpern ME Kimmel CB Nülsslein-Volhard C No tail (ntl) is the zebrafish homologue of the mouse T (Brachyury) gene Development 1994 120 1009 1015 7600949 10.1242/dev.120.4.1009

[B50] Stenkamp DL Frey R Mallory DE Shupe EE Embryonic retinal gene expression in sonic-you mutant zebrafish Dev Dyn 2002 225 344 350 10.1002/dvdy.10165 12412019

[B51] Park HC Kim CH Bae YK Yeo SY Kim SH Hong SK Shin J Yoo KW Hibi M Hirano T Miki N Chitnis AB Huh TL Analysis of upstream elements in the HuC promoter leads to the establishment of transgenic zebrafish with fluorescent neurons Dev Biol 2000 227 279 293 10.1006/dbio.2000.9898 11071755

[B52] Kimmel CB Ballard WW Kimmel SR Ullmann B Schilling TF Stages of embryonic development of the zebrafish Dev Dyn 1995 203 253 310 10.1002/aja.1002030302 8589427

[B53] Okada A Lansford R Weimann JM Fraser SE McConnell SK Imaging cells in the developing nervous system with retrovirus expressing modified green fluorescent protein Exp Neurol 1999 156 394 406 10.1006/exnr.1999.7033 10328944

[B54] Carreira-Barbosa F Kajita M Morel V Wada H Okamoto H Martinez Arias A Fujita Y Wilson SW Tada M Flamingo regulates epiboly and convergence/extension movements through cell cohesive and signalling functions during zebrafish gastrulation Development 2009 136 383 392 10.1242/dev.026542 19091770 PMC2687588

[B55] Campbell RE Tour O Palmer AE Steinbach PA Baird GS Zacharias DA Tsien RY A monomeric red fluorescent protein Proc Natl Acad Sci U S A 2002 99 7877 7782 10.1073/pnas.082243699 12060735 PMC122988

[B56] Lewis KE Eisen JS Paraxial mesoderm specifies zebrafish primary motoneuron subtype identity Development 2004 131 891 902 10.1242/dev.00981 14757641

[B57] Sun Z Su Y Meng A A novel zinc finger transcription factor resembles krox-20 in structure and in expression pattern in zebrafish Mech Dev 2002 114 133 135 10.1016/S0925-4773(02)00038-2 12175499

[B58] Topczewska JM Topczewski J Shostack A Kume T Solnica-Krezel L Holgan BL The winged helix transcription factor Foxc1a is essential for somitogenesis in zebrafish Genes Dev 2001 15 2483 2493 10.1101/gad.907401 11562356 PMC312789

